# Active-Passive Joint Acoustic Emission Monitoring Test Considering the Heterogeneity of Concrete

**DOI:** 10.3390/ma16247694

**Published:** 2023-12-18

**Authors:** Zhehan Chen, Tianjiao Miao, Tao Liu, Xuandong Chen, Aiping Yu

**Affiliations:** 1 College of Civil and Architectural Engineering, Guilin University of Technology, Guilin 541004, China; 2120210725@glut.edu.cn (Z.C.); 1020230752@glut.edu.cn (T.M.); 2120230888@glut.edu.cn (T.L.); 6616051@glut.edu.cn (X.C.); 2 Guangxi Key Laboratory of Geomechanics and Geotechnical Engineering, Guilin 541004, China

**Keywords:** concrete, active-passive joint acoustic emission monitoring, wave velocity, amplitude, time-of-arrival localization

## Abstract

The heterogeneity of concrete is a major challenge for acoustic emission monitoring. A method of active-passive joint acoustic emission monitoring considering the heterogeneity of concrete is presented herein, and the time-frequency-space multi-parameter response characteristics of active and passive acoustic emission signals were studied in relation to the damage evolution of concrete. This method provides an idea of evaluating the damage state of concrete more actively and quantitatively than traditional methods. The results show that the microscopic damage model of concrete based on the acoustic emission penetrating wave velocity and amplitude is in agreement with the damage process of concrete. The standard deviation of the wave velocity up to 1000 m/s and the change rate of the amplitude up to −0.66 can be adopted as two signs that the load of concrete reached 70% of the ultimate load. The time-of-arrival localization based on variable velocity was used to correct the acoustic emission localization results, and the localization accuracy was increased by 44.74%. The damage process of concrete undergoes diverse changes; that is, the distribution of damage changes from heterogeneous to homogeneous and then back to heterogeneous. Hence, it is necessary for researchers to consider the heterogeneity of concrete when using acoustic emission monitoring. The active-passive joint acoustic emission monitoring is an effective method.

## 1. Introduction

The damage evolution of concrete has always been of great concern. Traditional strategies rely on the continuity of materials and often treat the concrete material as homogeneous. Although this method can produce the desired results under certain conditions, the findings are often inconsistent, and some meaningful information about the heterogeneity of concrete is easily ignored [1,2]. Fang et al. [3] established a heterogeneous concrete model and found that the fracture evolution process of the heterogeneous model was more consistent with the results of a CT test than that of the homogeneous model. Zima et al. [4] found that Lamb waves propagate with different speeds in homogeneous and heterogeneous concrete models.

Acoustic emission (AE) technology is a passive monitoring technology, which can collect the instantaneous elastic waves released during concrete fracture in real time [5,6,7]. The fracture information carried by elastic waves is used to provide valuable clues for the dynamic damage of concrete [8,9,10]. A variety of AE characteristic parameters (energy, ringing count, hit count, etc.) related to concrete microcracks are often directly used to describe the accumulation, development, and acceleration of microcracks [11,12]. AE energy is the area of the AE waveform envelope, AE ringing count is the number of AE wave peaks, and AE hit is the number of waveforms that are collected. Chen et al. [13] found that the time-varying curves of cumulative AE hits and ringing counts have two obvious inflection points, which are related to the peak load and the boundary effect of concrete. In addition, cumulative AE energy can represent the changing trend of concrete fracture energy under different loading rates. Song et al. [14] thought that P-wave speed can be used as the precursor of concrete failure under cyclic tensile load. Some methods in signal processing (B-value, RA-AF, frequency-domain analysis, etc.) have been proposed and used to explain the fracture mechanism of concrete and identify the types of cracks [15,16,17,18,19,20].

The above research proved the feasibility of AE in identifying and diagnosing the damage of concrete, but the following two main problems still exist:

(1) The heterogeneity of concrete was not fully considered. AE waves are attenuated, dispersed, and diffracted in concrete, resulting in their distortion [21,22,23,24,25]. The complex propagation behavior of AE waves makes it difficult to consider the heterogeneity of concrete. In the current literature, numerical methods have been used to investigate the effect of heterogeneity on the propagation characteristics of AE waves. It was usually related to the size of specimen [26,27,28]. The effect of heterogeneity on the propagation characteristics of AE waves in small samples is generally considered to be negligible [29]. However, it is difficult to explain some experimental phenomena because the heterogeneity of concrete usually leads to uneven damage distribution, which further affects the propagation behavior of AE waves, resulting in greater errors. It is not conducive to the formation of a unified AE damage evaluation index. Mpalaskas et al. [30] and Tayfur et al. [31] emphasized that the heterogeneity of concrete can lead to incorrect AE results. For the same specimen, changing the position of AE sensors may result in different results. Li et al. [32] found that free water in a concrete medium influences its AE characteristics and ultrasonic propagation behavior. Obviously, the development of an active AE method considering the heterogeneity of concrete is of great significance for AE monitoring.

(2) The existing AE monitoring method is too dependent on the presence of loads. It is difficult for existing AE methods to actively detect the damage state of concrete under no load and constant load, and it is also difficult to quantitatively characterize the damage state of concrete. On the other hand, most of the AE microscopic damage models developed in the laboratory are dependent on the cumulative AE parameters [33,34,35]. Yu et al. [2] established a damage evaluation model of concrete using cumulative AE ringing counts and AE energy uniaxial compression. Gu et al. [36] established a damage constitutive model by AE energy, considering critical damage based on Weibull-distributed. To obtain the cumulative AE parameters, it is necessary to monitor the entire loading history of concrete. This makes AE monitoring too passive and time-consuming. Unexpected interruptions in testing are very detrimental to AE monitoring. This limits the application of AE. It is very important to find a method to evaluate the damage state of concrete actively, quantitatively, and timely.

This paper aims to propose an active-passive joint AE monitoring method considering the heterogeneity of concrete. Existing passive AE methods are the usual means of qualitatively evaluating the real-time developmental state of the damage of concrete. In contrast, active AE methods can quantitatively evaluate the damage state of concrete at a certain moment or stress level. Therefore, the active-passive joint AE monitoring method provides a more comprehensive understanding of the damage evolution mechanism of concrete. While exploring the active AE method, the influence of the heterogeneity of concrete in small scale samples on AE testing is discussed. The fundamental basis for the active AE detection method is that pores and cracks in concrete affect the propagation behavior of AE waves, and thus affect the AE information [37,38,39]. The artificially excited AE waves propagate through the sample, and the signal characteristics change, thus conveying information about the heterogeneity and damage state of concrete. Moreover, concrete samples with different water–cement ratios (w/c ratio) were tested by step-loading, and the active-passive joint AE tests were carried out under different loading levels in this paper. The time-varying and frequency-varying characteristics of active signals and passive signals are analyzed. The damage process of concrete is quantitatively explained by AE wave velocity and amplitude. The results indicate that the effect of concrete heterogeneity on AE characteristics should be considered even in samples of small size, and the wave velocity of active signals is used to correct the AE localization results, which turns out to be an effective approach.

## 2. Materials and Methods

### 2.1. Experiment Design

In the experiment, three groups of samples, A, B, and C, were prepared, and the w/c ratios were 0.58, 0.48, and 0.38, respectively. There were 6 samples in each group, numbered from 1 to 6. The size of the specimen was 150 mm ×150 mm × 150 mm. The mix ratios of the samples are shown in Table 1.

The cement was ordinary Portland cement (OPC). Fine aggregate is medium sand, and coarse aggregate is gravel with a maximum particle size of 20 mm. Cement, sand, and gravel were all produced in Guilin Xiangjiu Sand Field. All samples were made at the same time. After demolding, they were maintained under standard curing conditions for 28 days.

### 2.2. Testing System

**Figure 1:**
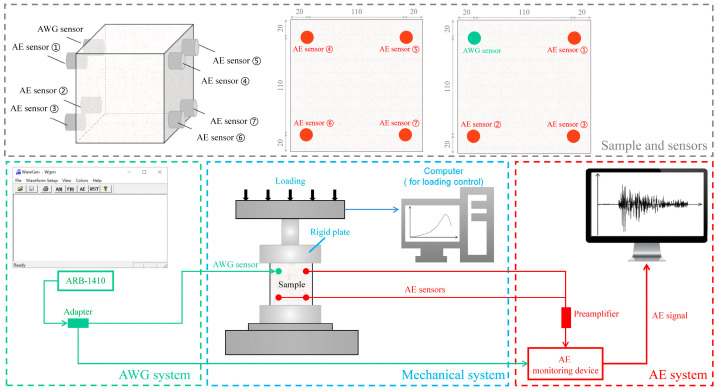
Test system diagram.

The test system is composed of mechanical, AE, and AWG systems, as shown in Figure 1. The mechanical system is SUNS universal testing machine. The AE system is Sensor Highway 3 AE device and is used to collect AE signals. AE sensor is PK15I narrow-band resonance sensor, and the operating frequency range is 100–450 kHz. The lower limit of frequency filtering is 20 kHz, and the upper limit is 1 MHz. The sampling frequency is 1 MHz. The AWG system is an arbitrary waveform generator (AWG), and the model is ARB-1410. The software WaveGen 13.0 can control the AWG to transmit AE signals of different frequencies. After initial testing, AWG signals of 120 kHz were relatively stable. Therefore, the signal of this frequency was selected for active AE test. Three AE and one AWG measuring points were set up on the left side of the sample, and four AE measuring points were set up on the right side of the sample. The field ambient noise and mechanical noise were less than 35 dB, so the AE threshold value was set to 40 dB.

### 2.3. Test Program

**Figure 2:**
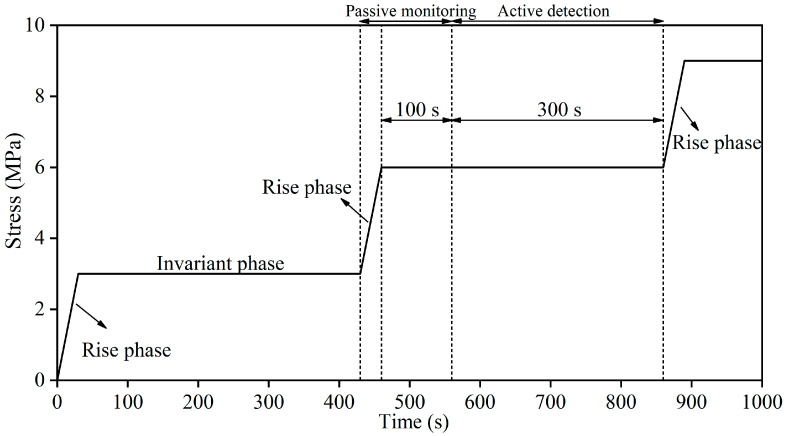
Test program diagram.

As shown in Figure 2, the test adopted the step-loading method. The loading process included Rise phase (*RP*) and Invariant phase (*IP*). The loading rate of *RP* was 0.1 MPa/s, and the *RP* lasted for 30 s. AE damage signals were collected throughout the *RP*, which is called passive monitoring. During the *IP*, which lasted for 400 s, the load remained unchanged. The samples were loaded to failure according to the above loading program. To eliminate the mutual interference between active and passive tests, the active test was conducted during the last 300 s of *IP*. The AWG was used to excite active signals, and the signals were received by 4#~7# AE sensors, which is called active detection.

### 2.4. Test Procedure

With an increase in load, microcracks in concrete will gradually develop. However, AE waves cannot propagate directly through cracks. When a crack is encountered, the propagation path of AE waves will change. The AE waves produced by AWG are also affected by cracks, and their characteristic parameters and frequency-domain characteristics will change. If this change can be captured, it can provide a basis for AE active detection. It is worth noting that the propagation mechanism of AE waves on the surface and inside the concrete is different. The microcracks are generated and developed inside the concrete. Microcracks have a more pronounced effect on AE waves propagating through the interior of the concrete (defined as penetrating waves). Therefore, it is more reliable to study the effect of microcracks on AE waves by using penetrating waves.

To further clarify these descriptions, AWG signals are called active signals, and the damage signals of samples are called passive signals.

### 2.5. Test Theory

#### 2.5.1. Microscopic Damage Model of Concrete

AE waves are generally assumed to propagate uniformly in a straight line through the material. Based on this assumption, the wave velocity of AE waves can be obtained using the following formula:

(1) $$v=\frac{l}{t_{1}-t_{0}},$$

where *v* is the average wave velocity of AE wave; *l* is the propagation distance of the wave; and *t*_0_ and *t*_1_ represent the arrival times of the transmitted and received waves, respectively.

Liu et al. [40] defined the damage variable of concrete as:

(2) $$D=1-\frac{\tilde{E}}{E_{0}},$$

where *D* is the damage variable, *E*_0_ is the elastic modulus of concrete in the initial state, and $\tilde{E}$ is the elastic modulus of concrete after damage.

The wave velocity of AE wave in medium is:

(3) $$v_{p}=\sqrt{\frac{E(1-\mu )}{\rho (1+\mu )(1-2\mu )}},$$

where *v*_p_ is the AE wave velocity, *E* is the elastic modulus of the medium, *μ* is the Poisson ratio of the medium, and *ρ* is the density of the medium.

Ignoring the changes of *μ* and *ρ* during the damage process of concrete, the microscopic damage model of concrete based on AE wave velocity can be obtained:

(4) $$D_{v}=1-{\left(\frac{{\tilde{v}}_{p}}{v_{p0}}\right)}^{2},$$

where *v*_p0_ and ${\tilde{v}}_{p}$ are the initial AE wave velocity and damaged AE wave velocity, respectively.

In fact, even for the same sample, the spatial evolution law of cracks observed at different measuring points is different. This makes the variation law of AE wave velocity also reflect a certain discreteness. This divergence can be demonstrated by using standard deviation. The standard deviation of the velocity of AE penetrating waves is obtained using Formula (5):

(5) $$S=\sqrt{\frac{\sum_{i=1}^{N}{\left(v_{i}-\bar{v}\right)}^{2}}{N}},$$

where *S* is the standard deviation of wave velocity, *v_i_* is the AE penetrating wave velocity, $\bar{v}$ is the average wave velocity, and *N* is the number of samples of penetrating wave velocity (*N* = 24).

Further, the microscopic damage model of concrete based on amplitude is obtained by using the method similar to Formula (6):

(6) $$D_{A}=1-{\left(\frac{{\tilde{A}}_{p}}{A_{p0}}\right)}^{2},$$

where *A*_p0_ and ${\tilde{A}}_{p}$ are initial AE amplitude and damaged AE amplitude, respectively.

The change rate of amplitude can be used to describe the trend of amplitude change. The change rate of amplitude is defined as:

(7) $$P=\frac{A_{i}-A_{j}}{\sigma_{i}-\sigma_{j}},$$

where *A_i_* and *A_j_* are the amplitudes obtained by the *i* and *j* measurements, respectively; $\sigma_{i}$ and $\sigma_{j}$ are the load on the sample at the *i* and *j* measurements, respectively.

#### 2.5.2. Time-of-Arrival Localization Based on Variable Velocity

**Figure 3:**
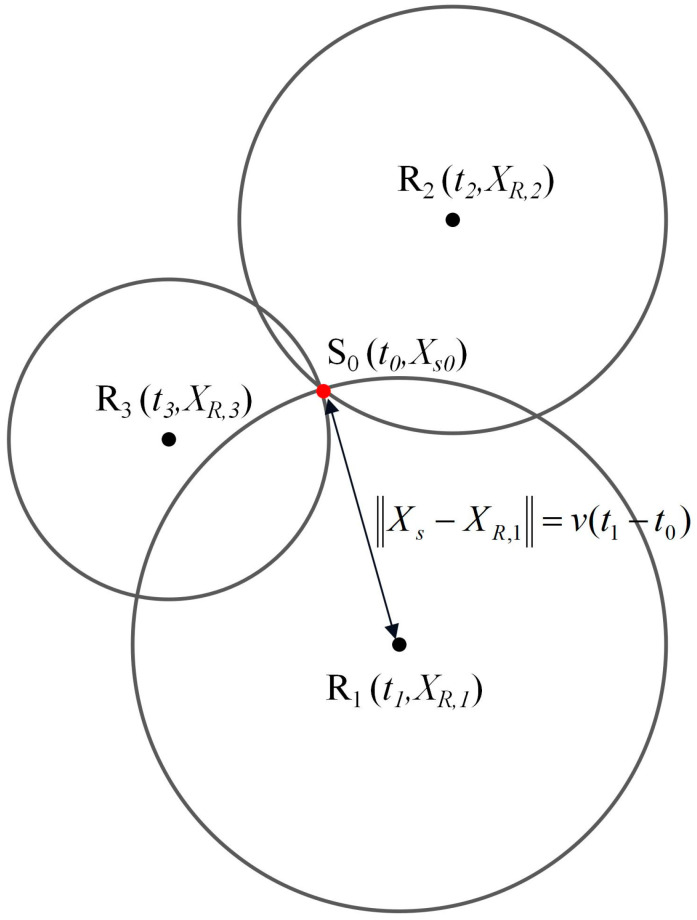
Diagram of time-of-arrival localization.

The basic idea of traditional time-of-arrival localization method is to estimate the location of AE source by the difference of arrival time of AE signal from different sensors and AE wave velocity. As shown in Figure 3, when an AE signal is received by multiple AE sensors (*R*_1_, *R*_2_, *R*_3_), AE wave velocity can be calculated using Formula (1). Then the source location is calculated using Formula (8):

(8) $$\left\|X_{S}-X_{R,i}\right\|-\left\|X_{S}-X_{R,j}\right\|=v\cdot (t_{i}-t_{j}),\forall i,j\in [1,2,\ldots ,n],$$

where *X*_S_, *X*_R,i_, and *X*_R,j_ represent the AE source location, the location of sensor *i*, and the location of sensor *j*, respectively.

In the traditional method, the wave velocity calculated using Formula (1) is considered to be constant. The results of this paper show that the development of cracks has a great influence on the AE penetrating wave velocity. Traditional methods do not consider this phenomenon, so it is easy to produce large errors. This paper proposes to correct the error of wave velocity related to the time-varying characteristics of AE wave velocity. By substituting the variable velocity into Formula (8), the localization results can be corrected. The above method is called the time-of-arrival localization based on variable velocity.

## 3. Results

### 3.1. Time-Varying Characteristics of AE Parameters

#### 3.1.1. Wave Velocity

**Figure 4:**
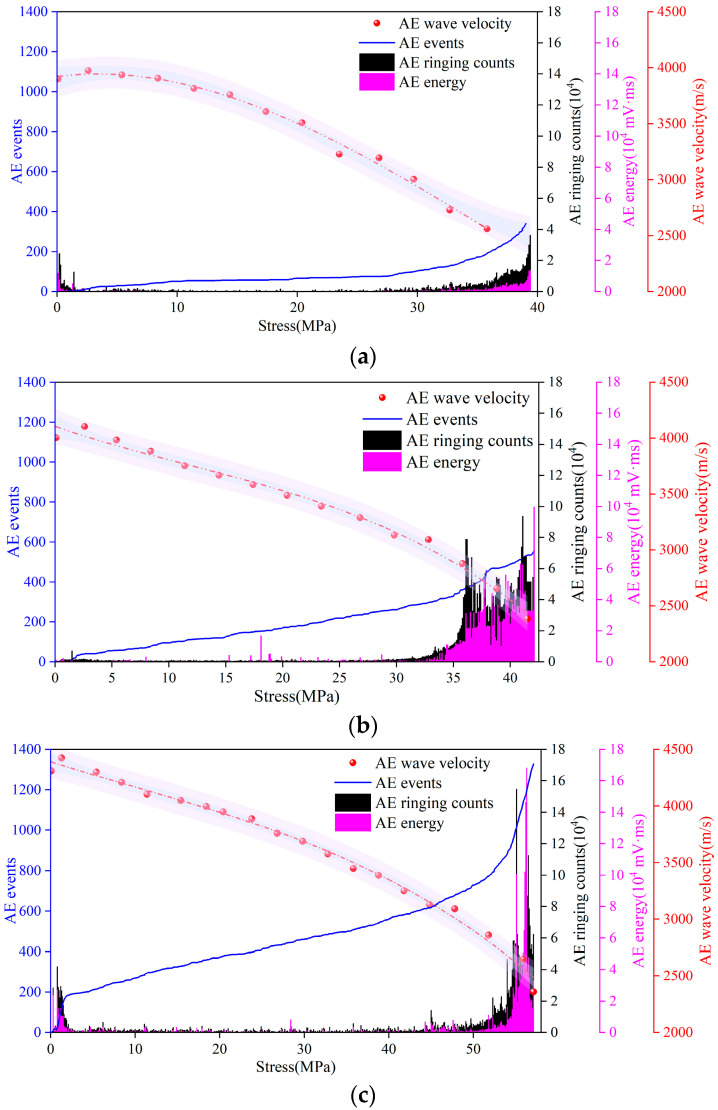
Wave velocity fitted curve and the relationship between stress and AE parameters for (**a**) Group A, (**b**) Group B, and (**c**) Group C.

As mentioned above, the initiation and development of microcracks will change the propagation path of AE waves. Therefore, the wave velocity calculated using Formula (1) will change. It should be noted that the wave velocity is usually constant in the same medium. The wave velocity described in this paper is the relative wave velocity calculated to reflect the effect of cracks on the AE wave propagation path (hereinafter referred to as wave velocity). The wave velocity is the average of the test results of 4#~7# sensors. Figure 4 shows the time-domain joint response relationship among AE wave velocity, passive signals, and stress in the damage process of the concrete.

It can be seen in Figure 3 that the concrete damage evolution process can be divided into three stages according to the time-varying characteristics of AE parameters:

- Compaction stage (stress level 0–10%): The initial microcracks within the concrete were closed under compression, resulting in AE events with certain ringing counts and energy. In this stage, the penetrating wave velocity increased slightly because the propagation distance of the penetrating wave was shortened to some extent by the closure of the original microcrack. The initial penetrating wave velocities of Groups A, B, and C were approximately 3899 m/s, 4044 m/s, and 4311 m/s, respectively. The peak penetrating wave velocities of Groups A, B, and C were approximately 3975 m/s, 4103 m/s, and 4425 m/s, respectively. The penetrating wave velocities of Groups A, B, and C were increased by 3.01%, 1.46% and 2.64%, respectively.
- Stable stage (stress level 10–70%): The closed initial microcracks opened under pressure and began to develop. New cracks started in the matrix and developed continuously. In this stage, the number of AE events continued to accumulate, and the penetrating wave velocity gradually decreased, indicating that the cracks were developing continuously. The AE ringing count and energy were low, indicating that the development of cracks was not drastic. Therefore, this stage was the AE quiet period.
- Unstable stage (stress level 70–100%): There was a qualitative change in the development of microcracks. The speed of crack development was accelerating. Many cracks crossed and aggregated and finally formed macroscopic cracks. The AE events increased sharply and carried high ringing counts and energies, indicating that the crack development was very intense. The penetrating velocity dropped sharply. The final penetrating wave velocity measurements of Groups A, B, and C were 2561 m/s, 2386 m/s, and 2359 m/s, respectively. Compared with the peak velocity, it was reduced by 35.57% (for Group A), 41.86% (for Group B), and 46.69% (for Group C). The smaller the w/c ratio, the larger the wave velocity loss ratio.

By fitting the wave velocity in Figure 4, the relationship between stress σ and wave velocity *v* can be obtained:

Group A:

(9) $$v=3948.72801+3.02704\sigma -1.18115\sigma^{2},R^{2}=0.99215,$$

Group B:

(10) $$v=4055.42762-13.45897\sigma -0.57768\sigma^{2},R^{2}=0.98677,$$

Group C:

(11) $$v=4354.65118-11.21413\sigma -0.36486\sigma^{2},R^{2}=0.98549$$

The correlation coefficients *R*^2^ of Formulas (9)–(11) are all greater than 0.98, and the accuracy of the results is high. The fitting curve is shown by the red dashed line in Figure 4.

#### 3.1.2. Microscopic Damage Model of Concrete Based on Wave Velocity

As mentioned in Section 3.1, the AE wave velocity first increased and then decreased during the damage process of the concrete. When the AE wave velocity increased, *D_v_* was negative, which was unreasonable. To avoid this contradiction, we took the maximum AE wave velocity during the damage process of the concrete as *v_p_*_0_. The AE wave velocity used for analysis here was the average of the data obtained from each set of six samples and four measuring points.

**Figure 5:**
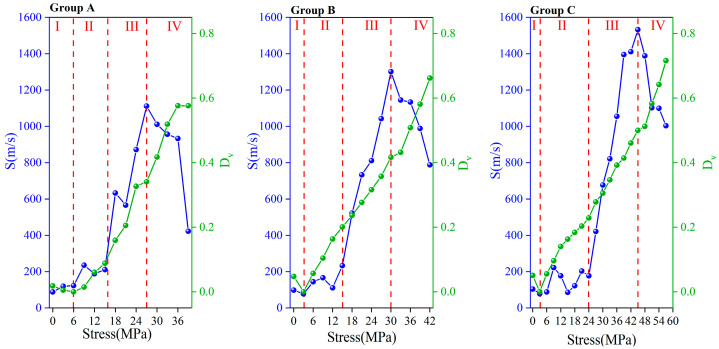
Concrete damage model *D_v_* and standard deviation of wave velocity.

As shown in Figure 5, *S* and *D_v_* in each group showed similar changes. *D_v_* represents the initial damage of the concrete before loading. During stage I (stress level 0–10%), the degree of damage of the concrete was reduced. *D_v_* decreased to 0, and there was little change in *S*. The maximum *S* values of groups A, B, and C were 123 m/s, 98 m/s, and 104 m/s, respectively. During stage II (stress level 10–40%), microcracks began to develop, and *D_v_* and *S* began to increase. *D_v_* and *S* grew slowly during this stage because the crack development was still very slow at this time. The maximum *S* values of groups A, B, and C were 235 m/s, 233 m/s and 222 m/s, respectively. During stage III (stress level 40–70%), the crack development accelerated. *D_v_* and *S* also accelerated, and their curves became steeper. The maximum *S* values of Groups A, B, and C were 1019 m/s, 1301 m/s, and 1532 m/s, respectively. At the end of this phase, the *S* reached its peak value. During stage IV (stress level 70 to 100%), crack development was very active, and *D_v_* increased at a faster rate until the sample failed. However, *S* decreased significantly during this stage. In the last test, *S* in each group decreased by 62.01% (for group A), 39.43% (for group B), and 26.79% (for group C) from the peak values. In summary, *S* reaching 100 m/s can be used as a harbinger of the beginning of microfracture development. When *S* reaches 1000 m/s or drops significantly, the concrete is about to be damaged.

#### 3.1.3. Amplitude

The amplitude can reflect the strength of the signal. The frequent occurrence of high-amplitude signals may be related to the macroscopic cracking of concrete. To facilitate observation, the amplitude of all passive signals was normalized.

**Figure 6:**
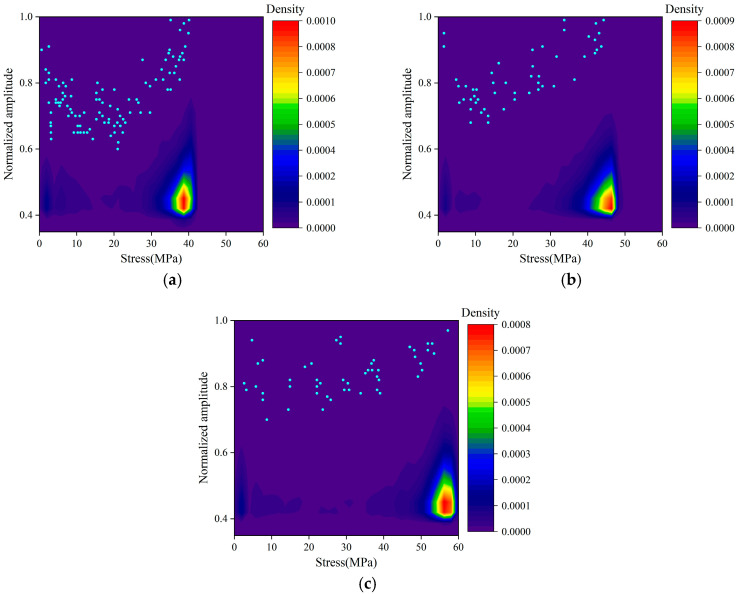
Amplitude density distribution of the passive signals for (**a**) Group A, (**b**) Group B, and (**c**) Group C.

Figure 6 shows the amplitude density distribution of passive signals and 50 points with the lowest density. Signals in the low amplitude range of 0.4–0.6 occupied the largest proportion. The density of low-amplitude signals increased with the increase in stress and reached a peak density near peak stress. The high-amplitude signals with amplitudes above 0.8 were mainly distributed in the later part of the loading process. At this point, it entered the unstable phase. The AE response associated with macroscopic cracking of the sample was very dramatic. This mirrors the AE ringing and energy surge described in Section 3.1.1 because, in general, the ringing count and energy of the high-amplitude signals was generally higher. The low-density distribution of high-amplitude signals was due to the small number of such signals. Compared with the low-amplitude signals throughout the whole damage process of the concrete, the number of high-amplitude signals was not dominant.

**Figure 7:**
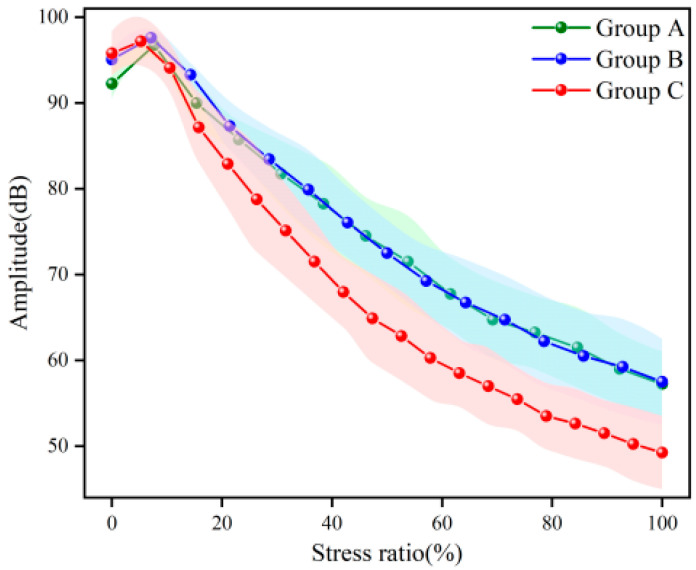
The amplitude of active signals. (The background color is error band).

Figure 7 shows the evolution of the amplitude of active signals. Similar to wave velocity, the amplitude showed a trend of first increasing and then decreasing. Before loading, the amplitude of the samples with a low w/c ratio was higher because the samples with a low w/c ratio had smaller porosity. The propagation distance of the penetrating wave was relatively short, and the attenuation degree was small. During the compaction stage, the amplitudes of groups A, B, and C increased by 4.88%, 2.68% and 1.44%, respectively. Unlike the wave velocity, the amplitude dropped more slowly during the late loading period than during the middle loading period. According to the quality factor Q theory, there is a negative exponential relationship between the amplitude and the propagation distance [41]. Therefore, the amplitude attenuation in this test resulted in a trend from fast to slow.

#### 3.1.4. Microscopic Damage Model of Concrete Based on Amplitude

Similarly, to avoid the unreasonable situation where *D_V_* is negative, the maximum AE amplitude during the loading process was taken as *A_p_*_0_. As mentioned in Section 3.1.3, the attenuation of the amplitude showed a tendency from fast to slow.

**Figure 8:**
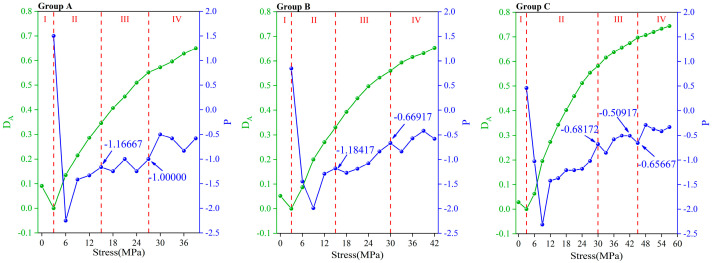
Microscopic damage model of concrete *D*_A_ and change rate of amplitude *P*.

As shown in Figure 8, *D*_A_ underwent a change of first decreasing and then increasing, which is similar to the change in *D_v_* in Section 3.1.2. The difference is that the ascending section of *D*_A_ showed a trend from steep to gentle. This corresponded with a change in *P*. When *P* was greater than 0, the amplitude increased. At this time, the sample was in the compaction stage, the damage degree was slightly reduced, and the *D*_A_ was reduced to 0. Subsequently, the crack began to develop, and *P* plummeted below 0, indicating that the amplitude began to decrease. The *D*_A_ began to rise, indicating that the damage was deepening. With the increase in load, *P* gradually increased, which means that the attenuation of the amplitude gradually slowed down. When the *P* reached −1.00000 (for group A), −0.66917 (for group B), and −0.65667 (for group C), the samples entered the unstable stage (Figure 8). This indicated that the sample was about to be destroyed. In summary, the changes in *D*_A_ and *P* were consistent with the damage process of the concrete.

### 3.2. Frequency-Varying Characteristics of AE Signals

Fast Fourier transform (*FFT*) was performed on the active and passive signals, and it was found that the spectrum characteristics of the two signals were obviously different.

**Figure 9:**
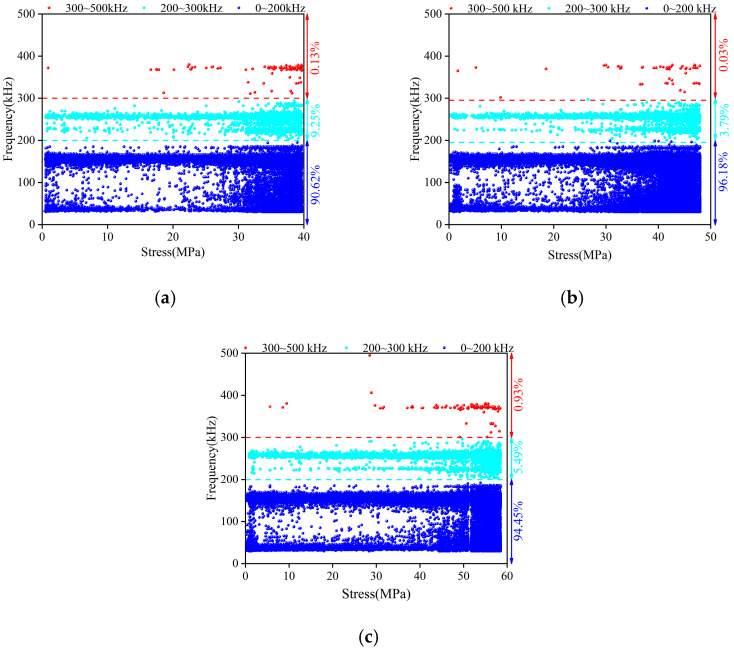
Main frequency of passive signals for (**a**) Group A, (**b**) Group B, and (**c**) Group C.

Figure 9 shows the main frequency for all passive signals. The passive signals had a wide frequency range within 0–500 kHz. Overall, the passive signals can be divided into low-frequency signals (0–200 kHz), medium-frequency signals (200–300 kHz), and high-frequency signals (300–500 kHz). The low-frequency and medium-frequency signals were almost all over the whole damage process of concrete. The proportions of low-frequency signals in groups A, B, and C were 90.62%, 96.18%, and 95.54%, respectively. The low-frequency signals occupied the dominant position regarding quantity. The high-frequency signals mainly existed during the late period of the loading process because the cracks during this period were very active, and it was easy to produce high-frequency signals. This corresponded to a surge of passive AE characteristic parameters in the later stage of loading process, as shown in Figure 3. It can be inferred that the high-frequency signals were related to the macroscopic cracking of the concrete. This feature can be used as a harbinger of concrete failure.

Further, taking group *A* as an example, the frequency spectrum signature of the signals in different damage stages were analyzed. Overall, the amplitude of the active signal was high, and the energy was concentrated near the main frequency. There was basically no energy distributed at the high-frequency bands. The main frequency and secondary frequency of the passive signals were messier, and there were even multiple secondary frequencies. The amplitude of the passive signal was lower, but the energy distribution range was significantly wider.

**Figure 10:**
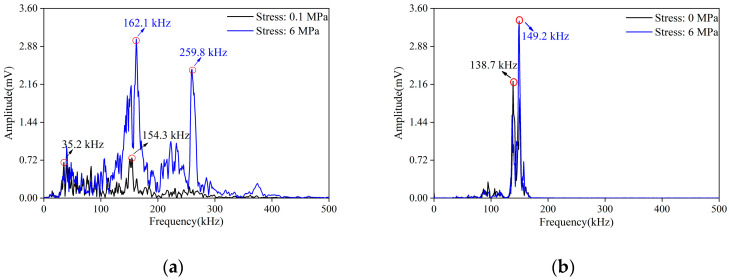
Spectrum diagram of active signals (**a**) and passive signals (**b**) during stage I.

During stage I, the active signal consisted of multiple peaks, and the microcracks were closed under pressure (Figure 10). With the increase in load, the initial damage of the sample decreased, and the load was not enough to produce nascent cracks. Therefore, the main frequency and the amplitude of each frequency band of the passive signal increased. At this stage, the attenuation of the AE signals decreased. The spectrum energy of the active signals shifted from 138.7 kHz to 149.2 kHz.

**Figure 11:**
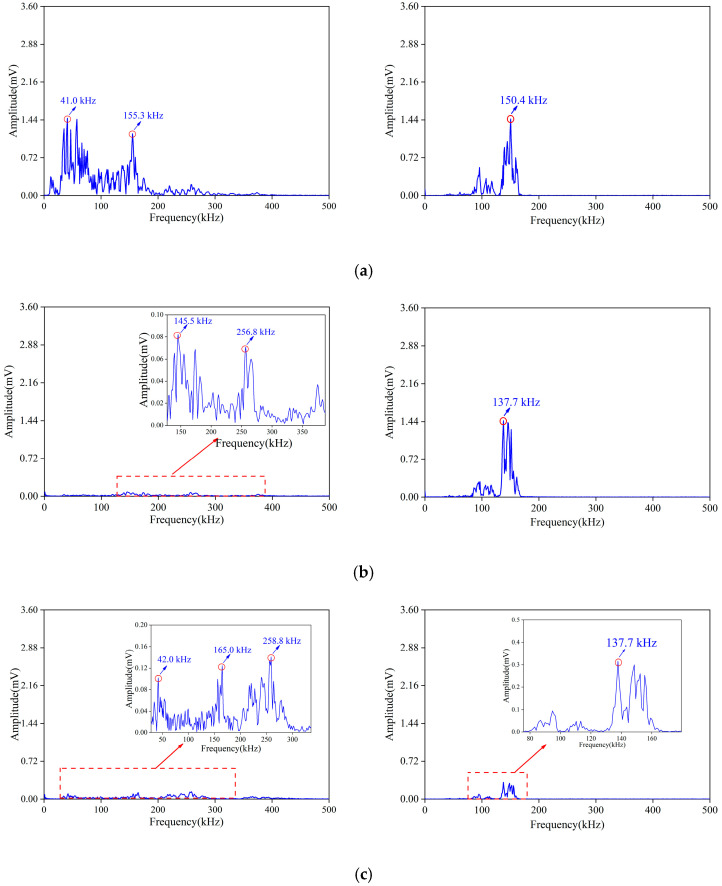
Spectrum diagram of active signals (**left**) and passive signals (**right**) during (**a**) stage II, (**b**) stage III, and (**c**) stage IV.

As shown in Figure 11, when the stress reached 12 MPa (stage II), initial cracks developed, and new cracks started. At this time, the fracture scale was small, and the development was slow. The spectrum energy of the active signal was still concentrated around 150.4 kHz, but the amplitude was reduced. When the stress reached 24 MPa (stage III), the fracture development accelerated. The main frequency of the passive signal moved from low frequency to medium frequency and the spectrum energy of the passive signal was concentrated in the middle-frequency band. The spectrum energy of the active signal shifted to 137.7 kHz. When the stress reached 39 MPa (stage IV), the sample was close to failure. The spectrum energy distribution of the passive signal was more complex, and the number of secondary frequencies increased. Due to the existence of many cracks, the attenuation of the AE signals increased and the amplitude of the active signal decreased.

### 3.3. Spatial Joint Response of Active and Passive Signals

**Figure 12:**
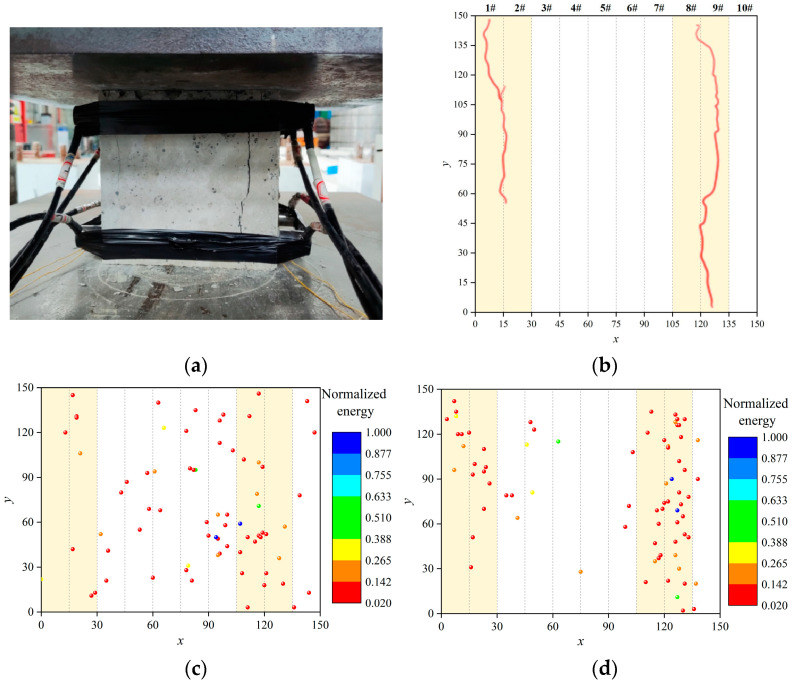
(**a**) Cracks in front facade of sample *A*_1_. (**b**) Crack bands. (**c**) Localization results before correction. (**d**) Corrected localization results.

In this paper, a two-dimensional localization method was adopted. Sample *A*_1_ was taken as an example to illustrate the correction process of the localization results. Figure 12a shows the crack morphology of the front façade when the sample is damaged. Taking the lower left corner of the facade of the sample as the origin, a plane coordinate system was established, and a crack morphology diagram was drawn, as shown in Figure 12b. The coordinate planes were divided into 10 longitudinal bands numbered 1#–10#. The bands where the cracks were located were defined as the crack bands. The AE localization points within crack bands were considered accurate.

The 467 AE events generated during the loading process of sample *A*_1_ were screened. The AE energy was used as a screening index because it was very sensitive to the macroscopic cracking of concrete. The AE energy of the 467 AE events was normalized, and the 76 AE localization points with an energy greater than 0.020 were analyzed. The localization results based on the traditional method are shown in Figure 12c. The AE localization points were dispersed in the plane. There were 30 AE events that were located in the crack bands with an accuracy of 39.47%. The AE localization results calculated using the time-of-arrival localization method based on the variable velocity proposed in this paper are shown in Figure 12d. The distribution of AE localization points in the plane was significantly changed. Most of the points converged towards the crack bands. There were 64 AE events located in the crack bands with an accuracy of 84.21%. The accuracy of the localization results increased by 44.74%. From the localization results, the new method proposed in this paper was closer to the actual location of the cracks. This shows that it is feasible to improve the accuracy of an AE source location by using the time-of-arrival localization method based on variable velocity.

## 4. Discussion

Generally, microcracks start and develop in heterogeneous layers. With the increase in microcrack density, microcracks interconnect with each other and form macroscopic fractures. Linear elastic fracture mechanics cannot fully explain this complex process [42,43]. In this study, the time-varying and frequency-varying characteristics of AE signals were used to visualize the damage evolution process of concrete. These characteristics provide a reference for AE to detect concrete actively. In addition, it was found that the damage evolution process of concrete is a changing process from heterogeneous to homogeneous and back to heterogeneous.

Take the wave velocity for example. If we treat the concrete sample as a homogeneous material, then the wave velocity captured by the 4#~7# sensors should be the same when there is no load. However, the 4#~7# sensors were different. As the load increased, cracks developed in the homogeneous layer. This development was random and had different effects on the sensors in different locations. Therefore, with the increase in load, the wave velocity captured by the different sensors will have a significant dispersion. This dispersion will increase with an increase in load. In short, if the concrete is regarded as a homogeneous material, the wave velocity will not be discrete in the initial state. As the load increases, the dispersion of the wave speed should increase.

However, in our experiments, we observed a different pattern. We used the standard deviation *S* of the wave velocity to measure the dispersion of the wave velocity and found that the dispersion of the wave velocity exists all the time. In the initial state, the *S* of groups A, B, and C were 88 m/s, 98 m/s, and 104 m/s, respectively. This showed that even in the initial state, the initial damage of concrete samples at different locations was different. This reflected the non-uniformity of the concrete sample. Even for the same sample, the wave velocity at different measuring points also showed discreteness. This is a satisfying detail because AE equipment usually calculates the AE source location based on wave velocity and then defines AE characteristics. The dispersion of the wave velocity showed the non-negligible effect of the heterogeneity of concrete on AE monitoring.

During the compaction stage, the wave velocity of the AE signal from different sensors increased by different degrees, whereas *S* decreased by different degrees. This indicated that the internal damage of the concrete sample was reduced during the compaction stage. The sample changed from heterogeneous to homogeneous. Similarly, with the increase in stress, the wave velocity decreased and *S* increased significantly. This indicated that the sample changed from homogeneous to heterogeneous.

To sum up, it is one-sided to regard small concrete samples as homogeneous materials, which was the case in previous tests. The heterogeneity of concrete should be considered in the exploration of concrete failure precursors and damage assessment methods and evaluated using AE technology. This also reflects the necessity of active-passive joint AE monitoring.

## 5. Conclusions and Prospects

The main conclusions can be summarized as follows:

1. During stage I, *D*_A_ and *D*_V_ decreased. Wave velocity and amplitude of the active signals increased. The main frequency and amplitude of the passive signals increased. During stage Ⅱ, *D*_A_ and *D*_V_ began to rise slowly. The wave velocity and amplitude of the active signals decreased. The energy of the passive signals decreased. During stage Ⅲ, *D*_A_ and *D*_V_ increased rapidly. The wave velocity of the active signals decreased significantly. The passive signals shifted to an intermediate frequency. During stage IV, the spectrogram of the active signals showed multimodal characteristics. The number of passive signals increased sharply, and the number of spectral peaks increased.
2. The microscopic damage model of concrete established in this paper based on the varying characteristics of the wave velocity and amplitude are in agreement with the damage evolution process of concrete. The standard deviation of the wave velocity reached 1000 m/s, and the change rate of amplitude reached −0.66; these changes can be used to signal that the load on the concrete has reached 70% of the ultimate load. Based on the relationship between the penetrating wave velocity and stress, the localization accuracy of the concrete was improved by 44.74%.
3. The damage evolution process of concrete is a changing process from heterogeneous to homogeneous and then back to heterogeneous. The dispersion of the penetrating wave velocity decreases first and then increases, and it is necessary to consider the heterogeneity of concrete even when monitoring samples of small size.

Future research could investigate the following:

1. Other factors that contribute to the heterogeneity of concrete, such as age, moisture content, aggregate size, and sand rate should also be considered.
2. The AE response of concrete under different work conditions should be considered. For example, it is necessary to analyze the damage sequence of concrete using fatigue mode, which is conducive to further improving the feasibility of proposed active-passive joint AE monitoring methods. A discussion of the Kaiser effect is indispensable during this step. We will complete this task in the next study.
3. This paper is a preliminary exploration of active-passive joint AE monitoring. In the future, this method will be used for the assessment of greater structures to enhance its applicability. Moreover, the application conditions of the active-passive joint AE monitoring method in RC structures should be further considered.
4. In addition, some non-contact detection methods, such as X-ray computed tomography and digital image correlation, can be combined with AE monitoring. Establishing the relationship between crack morphology, surface displacement, and the characteristics of active AE signals can further reveal the damage mechanism of concrete and improve the accuracy of AE results.

## Figures and Tables

**Table 1:** Proportions of concrete mix.

Group Code	W/C Ratio	Cement (kg/m^3^)	Water (kg/m^3^)	Sand (kg/m^3^)	Macadam (kg/m^3^)
*A*	0.58	458.72	266.06	534.05	991.80
*B*	0.48	500.30	240.10	545.60	1013.20
*C*	0.38	550.10	209.00	559.40	1038.80

## Data Availability

The data presented in this study are available on request from the corresponding author.
